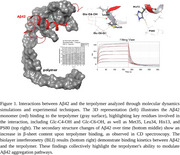# Elucidating the Interaction Dynamics Between a Novel Terpolymer and Aβ42: Unlocking Therapeutic Potential for Alzheimer's Disease

**DOI:** 10.1002/alz70855_103257

**Published:** 2025-12-23

**Authors:** Sako Mirzaie, Lily Yi Li, Chunsheng He, Jeffrey T. Henderson, Paul E Fraser, Xiao Yu Wu

**Affiliations:** ^1^ University of Toronto, Toronto, ON, Canada

## Abstract

**Background:**

Alzheimer's disease (AD), a progressive neurodegenerative disorder, is closely associated with the aggregation of amyloid‐beta (Aβ) peptides, particularly Aβ42. Toxic oligomeric forms of Aβ42 are implicated in synaptic dysfunction and cognitive decline, making them a key target for therapeutic interventions^1^. This study explores the interactions between a novel biodegradable terpolymer we developed for delivering imaging and therapeutic agents to the AD brain^2,3^ and Aβ42 to evaluate its therapeutic potential.

**Method:**

The biding affinity between Aβ42 and the terpolymer was evaluated using biolayer interferometry (BLI). Replica exchange molecular dynamics (REMD)^4^ simulation was performed to analyze conformational changes in Aβ42 upon binding to the terpolymer. Circular dichroism (CD) spectroscopy, transmission electron microscopy (TEM), and confocal microscopy were employed to monitor time course of structural transitions, aggregation patterns, and cellular interactions. SH‐SY5Y cells were used to assess the effect of the terpolymer on attenuating Aβ42‐induced cytotoxicity.

**Result:**

REMD simulations revealed rapid conformational transitions in Aβ42 upon terpolymer binding, shifting from random coil and α‐helical structures to stabilized β‐sheet‐rich states. CD spectroscopy demonstrated accelerated secondary structure transitions, corroborating the simulation findings. TEM showed that the terpolymer disrupted conventional Aβ42 aggregation patterns, resulting in smaller, irregular aggregates rather than typical long fibrils. Cellular assays indicated that the terpolymer significantly reduced Aβ42‐induced cytotoxicity, improving cell viability in the terpolymer treated groups. Confocal microscopy revealed cellular uptake of Aβ42‐terpolymer complexes, reducing extracellular toxicity and harmful interactions with cellular membranes.

**Conclusion:**

This study highlights the capability of the terpolymer in modulating Aβ42 aggregation dynamics, mitigating toxic oligomer formation and alleviating its neurotoxic effects. These findings present a promising approach for developing polymer‐based therapies targeting Alzheimer's disease, offering a novel pathway to combat neurodegeneration.

**References**

Okumura, H., *J Phys Chem B* **2023,** *127* (51), 10931‐10940.

Park E, et al. Advanced Science. 2023, 10(12):2207238.

Li, L.Y.; Park, E. et al. *Nanotoxicology* **2024**, 1‐20.

Li, L.Y.; Park, E. et al. Nanotoxicology 2024, 1‐20.